# Knowledge on Multi-Drug Resistant Pathogens, Antibiotic Use and Self-Reported Adherence to Antibiotic Intake: A Population-Based Cross Sectional Survey From Pakistan

**DOI:** 10.3389/fphar.2022.903503

**Published:** 2022-05-31

**Authors:** Hafsa Arshad, Ali Hassan Gillani, Jamshaid Akbar, Huda Abbas, Asma Bashir Ahmed, Syed Nouman Hassan Gillani, Rabeea Anum, Wenjing Ji, Yu Fang

**Affiliations:** ^1^ Department of Pharmacy Management and Clinical Pharmacy, School of Pharmacy, Xi’an Jiaotong University, Xi'an, China; ^2^ Drug Safety and Policy Research Center, School of Pharmacy, Xi’an Jiaotong University, Xi’an, China; ^3^ Centre for Health Reform and Development Research, Xi’an Jiaotong University, Xi’an, China; ^4^ Department of Pharmacy, Faculty of Biological Sciences, Quaid-I-Azam University, Islamabad, Pakistan; ^5^ Department of Pharmaceutical Sciences, School of Pharmacy, The Superior University, Lahore, Pakistan; ^6^ Department of Community Medicine Quaid-e-Azam Medical College, Bahawalpur, Pakistan; ^7^ Institute of Pharmaceutical Sciences, Karachi, Pakistan; ^8^ Allama Iqbal Medical College, Lahore, Pakistan; ^9^ Department of Pharmacy, Multan, Pakistan

**Keywords:** knowledge, antibiotic use, multi-drug resistant pathogens, population-based, adherence

## Abstract

**Objective:** Surveying public awareness of antibiotic use and antibiotics can identify factors relevant to the design of effective educational campaigns. The aim of this study was to evaluate the knowledge, attitudes, and practices related to antibiotic use and multidrug-resistant pathogens in the general population in Pakistan.

**Research Design and Methods:** Cross-sectional survey was conducted, using a 60 itemed structured questionnaire and recruited individuals by convenient sampling from the general population in the four provinces of the country. Descriptive statistics were used to evaluate the responses and the chi squared statistic was used to assess differences between groups.

**Results:** The response rate was 87.6% (6,684 out of 7,631 individuals). Half of the respondents had received at least one prescription of antibiotics in the 6 months preceding the survey. Knowledge about antibiotic use, (39.8%) individuals scored above the mean (≥3) showed good knowledge about antibiotic use. Urban residents and male showed significant higher knowledge (*p* < 0.001) about antibiotic use. Approximately 50% of the respondents correctly answered the question about antibiotic resistance. Of the 3,611 received antibiotics, 855 (23.7%) were indicated for cough, 497 (13.8%) for a sore throat, 335 (9.3%) for ear ache, 665 (18.4%) for a burning sensation during urination, 667 (18.4%) for wounds or soft tissue inflammation. MDR pathogen was perceived as an important topic by (4,010) 60.1% of respondents.

**Conclusion:** Participants were aware of the problem of multidrug-resistant pathogens and understood the responsibility of each individual to avoid the spread of these infectious agents.

## Introduction

The development of antimicrobial resistance (AMR) is a major public health concern in Pakistan (Bilal et al., 2021; Gillani et al., 2021) and worldwide (Aslam et al., 2018) AMR causes longer hospital stays, increased mortality, and substantial economic and intangible losses (Gillani et al., 2021). The rise in AMR has been positively associated with inappropriate handling and unnecessary use of antibiotics (Bilal et al., 2021), Several factors such as the physicians’ knowledge gap about current antibiotic recommendations inappropriate diagnosis, availability of antibiotic without prescription, incomplete antibiotic course and insufficient patient education by healthcare providers, overuse of antibiotics in livestock cause antimicrobial resistant at population level (Dhingra et al., 2020; Murray et al., 2022). Adherence to antibiotic treatment is essential in ensuring the therapeutic effects and to preventing the development of AMR (Aslam et al., 2018).

In some developing nations, Gram-negative bacteria are a major cause of neonatal sepsis (Yadav et al., 2018; Wen et al., 2021), and these microorganisms have developed increasing multidrug resistance (MDR) over the past decades (Uddin et al., 2021a) because of inappropriate and indiscriminate antibiotic use, easy availability, lack of regulations regarding antibiotic use, poor sanitation, and unsuccessful infection control in maternity wards. The appearance of a bacterial strain that is resistant to most available antibiotics is a major concern in Pakistan (Afzal, 2017; Gillani et al., 2017; Bhatti, 2020; Uddin et al., 2021b).

A considerable number of studies have shown that the general population plays a key role in spreading the AMR due to lack of awareness about antibiotics among population in Pakistan (Ching et al., 2019; Saleem et al., 2019). The World Health Organization has stated that appropriate awareness among both the public and healthcare providers is the key to averting AMR (WHO, 2014). Decreased antibiotic use could be achieved via multifaceted educational interventions to inform healthcare practitioners and the public of the harm of antibiotic overuse (Teixeira Rodrigues et al., 2019). Also, public’s current knowledge evaluation about antibiotics and the development of AMR could help to design policies and campaigns addressing these problems (Kosiyaporn et al., 2020). Few studies have focused on knowledge among the general population in Pakistan regarding antibiotic use (Akhund et al., 2019; Gillani et al., 2021) and none have focused on MDR. We therefore, conducted a study on knowledge of MDR pathogens in this population. The aim of study was to evaluate the knowledge, attitudes, and practices related to antibiotic use and multidrug-resistant pathogens in the general population in Pakistan.

## Methods

### Study Area

**FIGURE 1:**
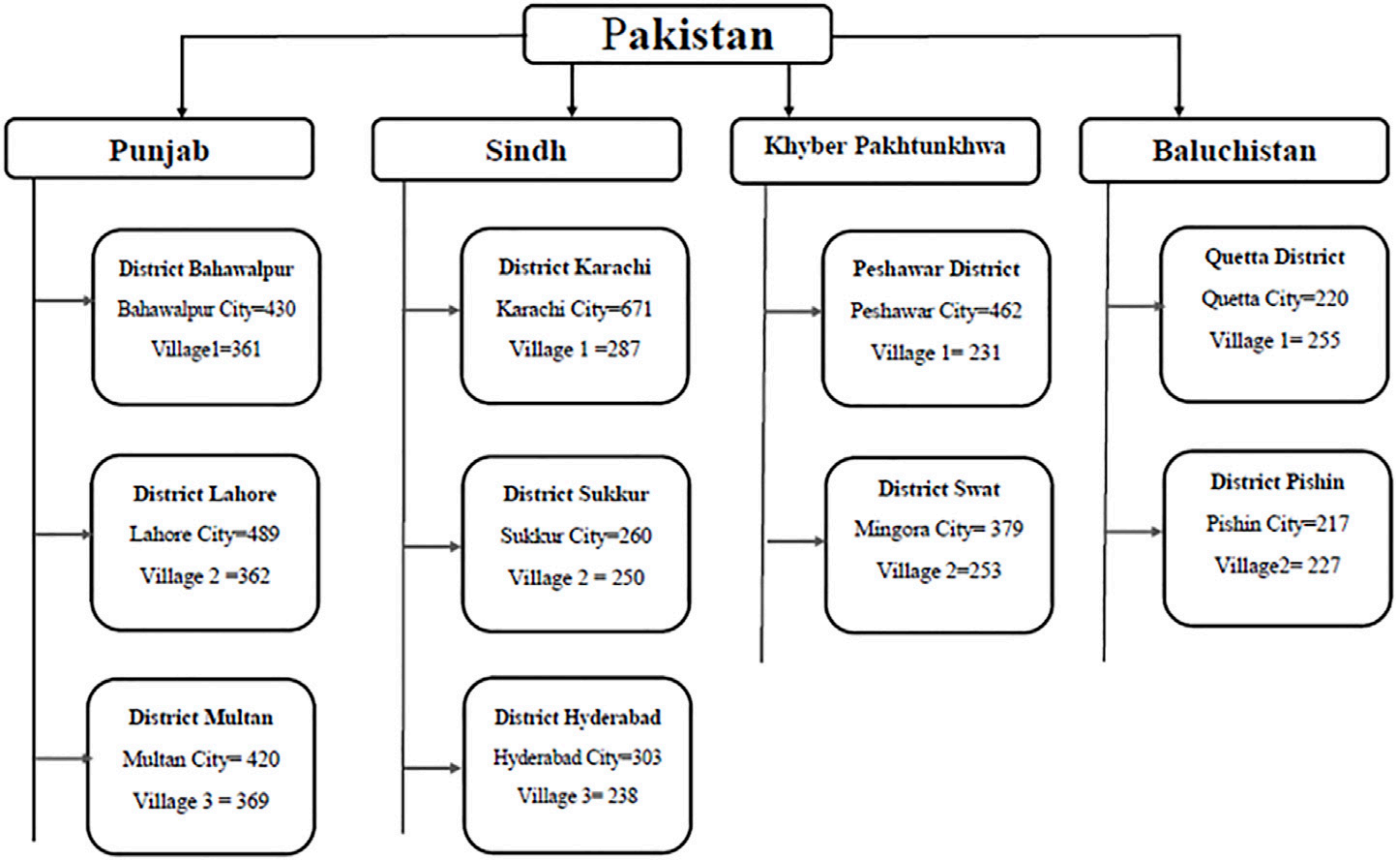
Selection of study area.

We sampled all four provinces of Pakistan (Punjab, Sindh, Khyber Pakhtunkhwa, and Baluchistan). The provinces consist of divisions, districts, tehsils (administrative areas containing towns), and villages. Three districts were randomly selected from Punjab and Sindh and two each from Khyber Pakhtunkhwa and Baluchistan. From each target district, we selected one city and one village. City was conveniently selected by the availability of data collectors. Figure 1 illustrates the cities and villages selected and the corresponding numbers of respondents.

### Study Participants and Recruitment

**FIGURE 2:**
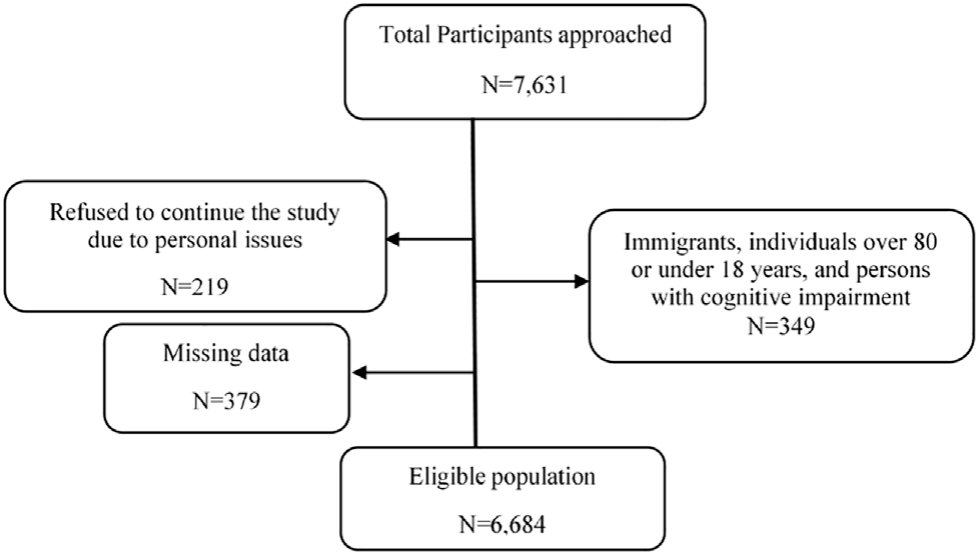
Fears of individuals regarding MDR pathogen.

We performed a cross-sectional survey in the four provinces of Pakistan. We used convenient sampling and try to collect data from maximum participants during May to November, 2020. Total 7,631 participants approached in the study during this time frame. Participants who were Immigrants, individuals over 80 or under 18 years, and persons with cognitive impairment (N = 479), participants initially participated in the study and during study refused to continue the study due to personal issues (lack of time, information sharing hesitation and others) (N = 219), and missing data from survey (N = 379) were excluded from the study. Finally, sample of 6,684 participants was included in the study. (Figure 2). Individuals were approached in shopping malls and in pharmacies, educational institutions, bus stations, train stations, and households. Self-administered questionnaires were filled in and collected. Interviews were conducted with individuals who were not literate. Individuals were made aware of the purpose of this study.

### Training of Data Collectors

Sixteen data collectors were trained to conduct the research in the provinces. Most data collectors were pharmacy students in their final year of study, who were supervised by teachers from the local university. Training covered the following aspects: 1) presenting a concise introduction of the study rationale to respondents; 2) conducting face-to-face interviews; 3) coping with difficulties in data collection, such as lack of cooperation. The training lasted 3 days and included a demonstration by the primary researcher.

### Questionnaire

The knowledge, attitudes, and practices regarding antibiotic use covered four topics: 1) exposure to antibiotics, evaluated by two questions about symptoms and prescription in the preceding 6 months. 2) Knowledge about antibiotics, evaluated by responses to seven statements related to correct identification of antibiotics and to opinions regarding antibiotic use in general. 3) Attitudes and practices related to antibiotic use, assessed by seven items concerning how participants generally obtained antibiotics and whether they were concerned about antibiotic resistance. 4) Whether participants requested antibiotics for personal use, how they used antibiotics, and whether they developed side effects during use (six items). The questionnaire used in this study differed in its contents from those of previous studies (Akhund et al., 2019; Gillani et al., 2021). The second part of the questionnaire addressed MDR pathogens: 1) awareness of MDR pathogens and past exposure (seven items). 2) Knowledge about MDR pathogens, assessed by questions and statements about infection routes, spread, and treatment of MDR pathogens (five items). 3) Attitudes about MDR pathogens, assessed by asking participants about their feelings with respect to contracting an MDR organism, about MDR pathogens as a public health problem, and their opinion regarding who is responsible for the control of their spread (10 items). 4) Reaction to close contact with an infected person, assessed using two case scenarios. The case scenarios were presented to assess how participants would deal with carriers of MDR pathogens.

Case 1: Let us suppose that your neighbor, an elderly person residing alone, requires some help and you have been shopping for him for a couple of months. After a hospital stay, he tells you that he has become infected with a hospital-acquired pathogen. How would you behave with him/her?

Case 2: Let us suppose that your coworker, with whom you share an office and some office items, tells you after a hospital stay that she has become infected with a hospital-acquired pathogen. How would you behave with him/her?

Each case scenario was associated with eight statements, and response options were “strongly agree,” “agree,” “somewhat disagree,” and “disagree.”

In addition, demographic information (age, sex, education, locality, and marital status) was also collected and included in the analysis. The questionnaire was modified slightly from a previous study (Akhund et al., 2019) according to our study protocol and translated in to national language. tResponses were back-translated into English. Construct validity and content validity of questionnaire was established by extensive literature either the designed tool was measuring the actual research question which we want to measure. Face validity was ensured when expert researchers looked at the items in the measuring tool and gave their expert opinions. Designed questionnaire was reviewed and evaluated by the research committee of four professors of pharmacy background to assess appropriateness of each question to be measure. After discussion with researchers their expert feedback was used to edit the instrument accordingly. Internal consistency was measure by Cronbach’s alpha. Cronbach’s alpha of questionnaire was 0.768 showed the reliability of research tool was good. Before the start of data collection, a pilot study was conducted in each area to check the accuracy of the wording and comprehensiveness. The data obtained in the pilot study were not included in the final analysis.

### Data Analysis

Descriptive analysis was performed for the demographic variables. To analyze the knowledge about antibiotics, each correct response was awarded one point. For a positive question, the responses “strongly agree” or “agree” were calculated as one point and “do not know,” “disagree,” or “strongly disagree” were calculated as zero. For a negative question, “disagree” or “strongly disagree” were calculated as one point and “strongly agree” or “agree” and don’t know were calculated as zero. A cumulative score of the seven knowledge items on antibiotics and the four items on MDR was derived in the same manner. The cut point of ≥3 is considered to be good knowledge for the knowledge about antibiotic as it was also chosen in the previous studies that those who scored more than mean were considered in good range. A complete questionnaire is subjected to the analysis those with the missing data were excluded in screening phase. Percentages for each item were calculated and the chi squared statistic was used to check significant differences between demographic groups. *p* < 0.05 was considered significant.

### Ethics Approval

The research was carried out in accordance with the tenets of the Declaration of Helsinki and was approved by the Medical Research Ethics Committee of Xi’an Jiaotong University, Shaanxi, China, approved reference number (XJTMD11-2020). Respondents who were literate provided a signed consent form and for those who were not literate, the data collector signed the form on their behalf after explaining the content of the form.

## Results

### General Characteristics of the Respondents

Out of 7,631 individuals who were approached for the study, 6,684 (87.6%) completed questionnaire items on antibiotic use and MDR pathogens. The mean age (± standard deviation) of the respondents was 32.97 (10.8) years, 4,746 (71.0%) were male, 772 (11.5%) had education above post-graduation and 2,833 (42.4%) resided in rural areas (Table 1).

**TABLE 1:** Demographic details.

Variable	Number (%)
Age (Mean ± SD) (years)	
32.97 ±10.8	
Gender	
Male	4,746 (71.0)
Female	1938 (29.0)
Residence	
Rural	2,833 (42.4)
Urban	3,851 (57.6)
Education	
Primary or below	1749 (26.2)
Secondary school	1,338 (20.0)
High school	1,558 (23.3)
College/university	1,267 (19.0)
Post graduation or above	772 (11.5)
Marital Status	
Single	3,439 (51.5)
Married	3,245 (48.5)
Monthly Household Income (RS)	
<15,000	2,398 (35.9)
15,000–3,000	2099 (31.4)
30,001–50,000	1,047 (15.7)
>50,000	1,140 (17.0)
Province	
Punjab	2,431 (36.4)
Sindh	2009 (30.1)
KPK	1,325 (19.8)
Baluchistan	919 (13.7)

### Knowledge About Antibiotics and MDR Pathogens

The mean (± standard deviation) of the knowledge score of antibiotic use was 2.51 (1.80) and 2,260 (39.8%) individuals scored above the mean (≥3). Few participants provided correct answers to the questions about antibiotics, and those tended to be urban residents and male showed significant higher knowledge (*p* < 0.001). Approximately half of the respondents correctly answered the question about antibiotic resistance. Supplementary Table S1 lists details about respondents and antibiotic use and Supplementary Table S2 responds to the answers of the participants regarding the attitude. The mean (± standard deviation) of the MDR knowledge score was 1.98 (1.86). Almost one third (32.7%) of participants correctly answered the question regarding whether MDR pathogens could only infect them in a hospital, and tended to be urban residents and male. Supplementary Table S3 lists details about respondents and MDR pathogens. Supplementary Tables S1, S2, S3 are presented in supplementary material.

### Exposure to Antibiotics and MDR Pathogen

**FIGURE 3:**
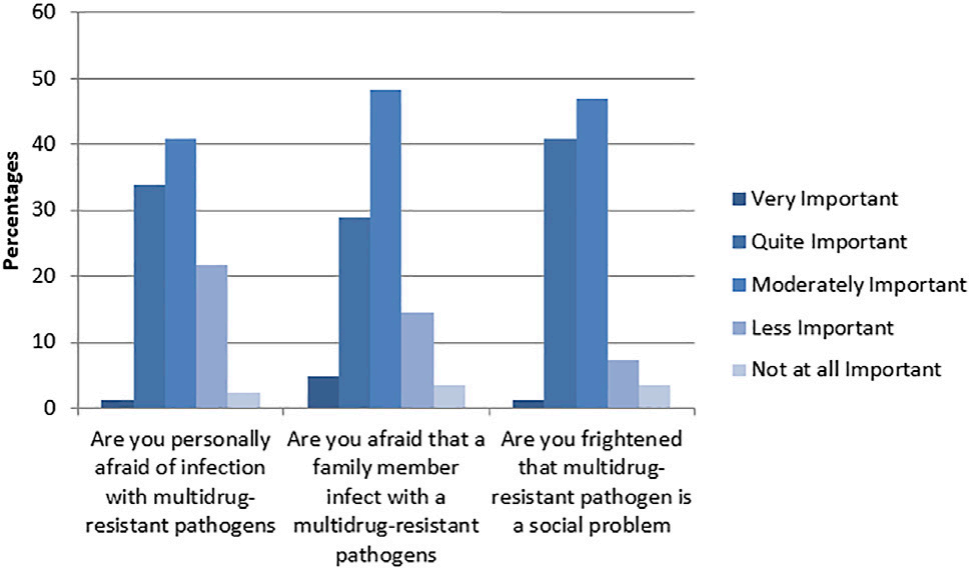
Fears of individuals regarding MDR pathogen.

Fifty-four percent of respondents (3,611) reported that they were prescribed antibiotics within the preceding 6 months, with a significantly higher proportion of participants from Sindh (1,274 or 35.3%) than Khyber Pakhtunkhwa (13.1%, *p* < 0.001). Of the 3,611 who received antibiotics, 855 (23.7%) were indicated for cough, 497 (13.8%) for a sore throat, 335 (9.3%) for ear ache, 665 (18.4%) for a burning sensation during urination, 667 (18.4%) for wounds or soft tissue inflammation, and 677 (18.5%) for other reasons. Almost two thirds (2,332 or 64.5%) of those who were prescribed antibiotics stopped the therapy before completion, because 433 (18.6%) felt better, 544 (23.3%) were worried about side effects, 78 (3.3%) experienced a side effect, 455 (19.5%) forgot to take the medication, 211 (9.0%) experienced “too much stress,” and 611 (26.2%) for other reasons. Almost two thirds 4,211 (63%) of all participants had heard of MDR pathogens, 7.3% 488) reported that they knew an individual who had tested positive for an MDR pathogen, and 12% (802) reported that they themselves had tested positive for an MDR pathogen once in their lifetime. Figure 3 illustrates the fears of respondents regarding MDR pathogens.

### Attitudes and Practices Regarding Antibiotic Use and MDR Pathogens

Almost half of the participants (47.6% or 3,181) reported sharing medications with family members and 35.8% 2,393) reported that they had antibiotics at home to use when necessary. MDR pathogen was perceived as an important topic by (4,010) 60.1% of respondents (Table 2). In terms of the responsibility to halt the spread of MDR pathogens in the healthcare sector, 30.9% (2065) of the participants thought that it rested with healthcare workers, 2,239 or 33.5% thought that it was the responsibility of everyone, and 1,430 or 21.4% believed that political administrators were responsible for halting the spread. Responsibility for reductions in antibiotic use in livestock breeding was attributed to farmers (23.6% or 1,564), political administrators (1,430 or 21.4%), and consumers (795 or 11.9%). One quarter of respondents 1,678 (25.1%) agreed that they would be willing to spend more money on meat if this could reduce antibiotic use in livestock (Table 3)

**TABLE 2:** Causes you think important for distributions of MDR pathogens.

Statement	Very important	Quite important	Moderately important	Less important	Not important	Don’t know
Improper antibiotic intake in population	410 (6.1)	1,403 (21.0)	829 (12.4)	745 (11.1)	825 (12.3)	2,472 (37.0)
Improper use of antibiotics in livestock breeding	409 (6.1)	1,234 (18.5)	663 (9.9)	742 (11.1)	743 (11.1)	2,893 (43.3)
Lacking hygiene in medical sector in general	409 (6.1)	1811 (27.1)	1,075 (16.1)	745 (11.1)	826 (12.3)	1818 (27.1)
Lacking hand hygiene from medical healthcare workers	83 (1.2)	249 (3.7)	744 (11.1)	1,481 (22.2)	2,145 (32.1)	1982 (29.7)
Lacking hand hygiene in society	497 (7.4)	577 (8.6)	662 (9.9)	1,401 (21.0)	1,402 (21.0)	2,145 (32.1)
Lacking bed capacity in hospitals	409 (6.1)	904 (13.5)	497 (7.4)	580 (8.7)	743 (11.1)	3,551 (53.1)
Too less effective medicaments	402 (6.0)	1,653 (24.7)	826 (12.4)	745 (11.1)	745 (11.1)	2,315 (34.6)

**TABLE 3:** Different stakeholders’ role to control the antibiotic resistance.

Statement	Totally agree	Agree	Rather disagree	Disagree	Don’t know
Politicians are responsible for reducing the use of antibiotics in livestock breeding	477 (7.1)	953 (14.3)	1830 (27.4)	1,591 (23.8)	1833 (27.4)
Farmers are responsible for reducing the use of antibiotics in livestock breeding	479 (7.1)	1,033 (15.5)	1988 (29.7)	1830 (27.4)	1,354 (20.3)
Consumers are responsible for reducing the use of antibiotics in livestock breeding	793 (11.9)	0 (0.0)	2,228 (33.3)	1751 (26.2)	1912 (28.6)
I am willing to spend more money for meat (comparable with costs for organic products) if this leads to reducing the use of antibiotics	239 (3.6)	1,437 (21.5)	1,669 (25.0)	1,511 (23.6)	1828 (27.3)
**The following three questions concerns who is responsible to limit the spread of antibiotic resistant pathogen on the healthcare system**					
Each individual has a responsibility, to correctly take antibiotics, to reduce the spread of multidrug-resistant pathogens	639 (9.6)	1,595 (23.9)	1,430 (21.4)	1,431 (21.4)	1,589 (23.8)
Doctors and health care staff are responsible for reducing /combating spread of multidrug-resistant pathogens in the healthcare system	555 (8.3)	1,510 (22.6)	1912 (28.6)	1,435 (21.5)	1,272 (19.0)
Politicians are responsible for reducing/combating spread of multidrug-resistant pathogens in the healthcare system	478 (7.1)	954 (14.3)	1991 (29.8)	1,430 (21.4)	1831 (27.4)

### Case Scenarios

**FIGURE 4:**
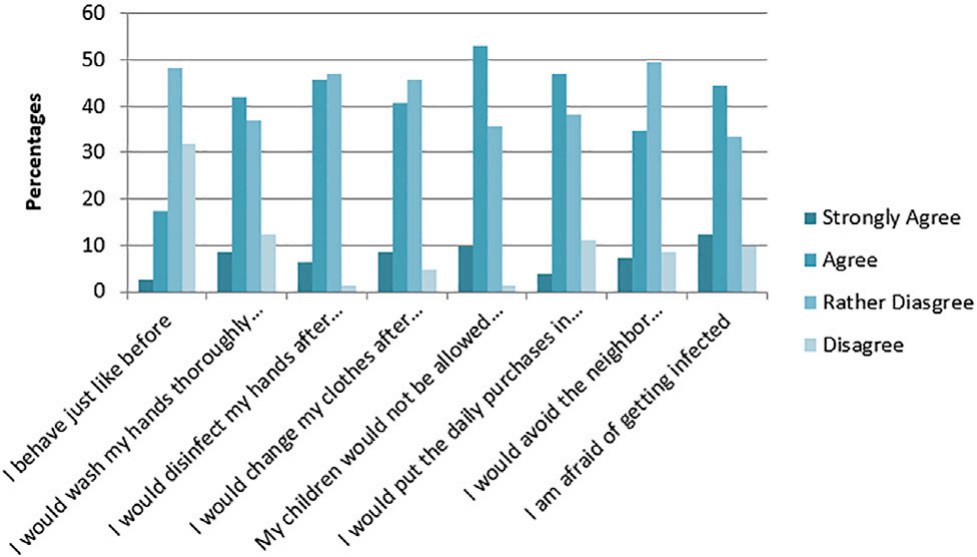
Response to case scenario one.

**FIGURE 5:**
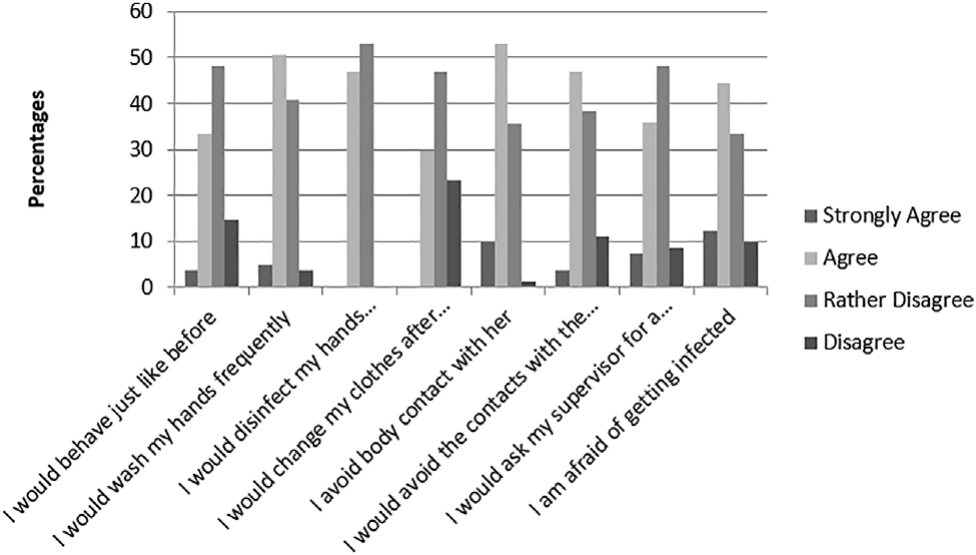
Responses to case scenarios 2.

The first scenario involved an aged neighbor hospitalized with an MDR pathogen infection and the second scenario involved a coworker testing positive for an MDR pathogen. For the first scenario, half of the respondents demonstrated good hygiene practices: 50.6% would wash their hands, 51.8% would disinfect their hands, 49.3% would change clothes after interacting with the neighbor, and 41.8% would avoid the neighbor completely (Figure 4). For the second scenario, 55.6% would wash their hands, 46.9% would disinfect their hands, 43.2% would avoid the coworker completely, and 29.6% would change clothes after interacting with the coworker (Figure 5).

## Discussion

In this study, we assessed the knowledge and opinions of the general public in Pakistan about antibiotic use and MDR pathogens by interviewing the participants. Individuals showed poor knowledge of antibiotic use and poor medication adherence. Similarly, knowledge about MDR pathogens was scant, but fear of the effects of MDR pathogens was more pronounced.

More than half had been prescribed antibiotics in the past 6 months, a lower percentage reported for the United Kingdom (38%) and Germany (Holstiege et al., 2020). The main indications for prescription in the present study were cough and sore throat, similar to the indications cited in a report from Germany (Holstiege et al., 2020) and a previous study in Pakistan (Gillani et al., 2021). Upper respiratory infections are most commonly caused by viruses, and international health agencies recommend restrictive measures in antibiotic use so as to prevent AMR (Shaikhan et al., 2018; Gulliford et al., 2019). The knowledge scores for antibiotic use were poor, lower than scores in the previous study in Pakistan (Gillani et al., 2021) but similar to those of studies in other developing nations (Seid and Hussen, 2018; Teixeira Rodrigues et al., 2019).

In the present study, 63% of participants had heard about MDR pathogens, lower than the percentage reported by a study in Germany (94.9%) and in Scotland (86%) (Raupach-Rosin et al., 2019; Tonna et al., 2019). Very few of our respondents reported having been diagnosed with MDR pathogens (12%) or knowing someone infected with an MDR pathogen (7.3%), unlike the 42.7% reported to be acquainted with a person diagnosed with MDR pathogen in Germany or the 32.0% reported to be acquainted with a person diagnosed with methicillin-resistant *Staphylococcus aureus* in Scotland (Raupach-Rosin et al., 2019; Tonna et al., 2019).

We did not find a significant difference in antibiotic use scores between urban and rural residents, in contrast with the previous study in Pakistan (Gillani et al., 2021) or with outcomes reported by a study in China (Wang et al., 2019), both of which reported poor knowledge of antibiotic use among rural residents. Also, it was observed in the study from Croatia where urban parents were more aware (Farkaš et al., 2019) In addition, we did not find significant differences in our study population in terms of education level and knowledge, in contrast with findings from the study in Germany (Raupach-Rosin et al., 2019). Our study however suggested the gender significant difference in most of the knowledge items which is in consistent with the previous results in Italy (Bianco et al., 2020). This suggests that information for the general public should be tailored for comprehensibility. Gaps in specific knowledge about the implications of MDR pathogens and existing treatment options have been observed before (Tonna et al., 2019; McClelland et al., 2022). Hence, targeting information to the population and using appropriate media are essential for increasing the public’s knowledge of proper antibiotic use and MDR pathogens.

More than half of the participants reported that they were personally concerned about infection with a MDR pathogen, but the answers to the case scenarios illustrated that the majority of individuals exhibited reasonable and non-stigmatizing behavior toward carriers of MDR pathogens in their vicinity. This finding is remarkable, considering that more than half of the participants had stated that they were fearful of contracting MDR pathogens. The percentage of respondents that reported they would avoid the infected person was higher than that reported by Raupach-Rosin and colleagues (Raupach-Rosin et al., 2016). A large number of respondents considered healthcare personnel and political administrators responsible for controlling MDR pathogens, similar to a finding reported previously (Gilbert and Kerridge, 2019).

### Strengths and Limitations

The strength of our study is the comparably large sample and the fact that the study population was sampled from the general public. However, because the knowledge of antibiotics and MDR pathogens was assessed by closed-ended questions, respondents may have selected the most favorable answers rather than accurate ones, and therefore, a qualitative approach might be more suitable to reveal misconceptions. Our study faced some limitations as well such as we selected the districts randomly and targeted the cities, village and participants from the city conveniently. Also, that participants may have given socially desirable answers, rather than actual practices.

## Conclusion

General knowledge about antibiotic use and MDR pathogens was poor in the population we sampled. However, there was a high awareness about antibiotic resistance as a public health problem, although only a few respondents reported to have been personally affected by MDR. Therefore, information about the implications of MDR pathogens should be made available for the general public. Information campaigns about correct antibiotic use could increase knowledge, thereby improving appropriate practices in antibiotic use and resistance in both humans and livestock. The data in this study provide new information and may serve as the basis for development of interventional programs and policies.

### Recommendations

We recommend strict government policies should be implemented at hospital level and community pharmacy level to prescribe and dispense antibiotics in the presence of qualified physician and pharmacist. Standard guidelines and proper trainings should be provided to physicians and other health care providers to promote the safe prescribing of antibiotics to avoid the antimicrobial resistance. Community based interventions and programs should be initiated to increase the awareness about antibiotic resistant among general population. Government should increase the awareness and initiate the campaigns on electronic media for safe use of antibiotic in general population. Collective efforts of government, stakeholders, health care providers, patients, researchers, pharmaceuticals companies, policy makers and drug regulatory bodies will help to combat the life threatening scenario of antimicrobial resistant around the globe.

## Data Availability

The original contributions presented in the study are included in the article/Supplementary Material, further inquiries can be directed to the corresponding authors.
