# Supplementary material for: Knowledge on Multi-Drug Resistant Pathogens, Antibiotic Use and Self-Reported Adherence to Antibiotic Intake: A Population-Based Cross Sectional Survey From Pakistan
Source: Front Pharmacol. 2022 May 31;13:903503. doi: 10.3389/fphar.2022.903503 (PMC9194673; doi:10.3389/fphar.2022.903503)
Supplement: Supplementary file 1 [file DataSheet1.docx]

**Table S2** **Distribution of knowledge regarding antibiotics use according to general characteristics of the study population**

| **Statement** | **Gender** | |  |  | | **Residence** | | | **Education** | | | | | | | | | | | | | |  | |  |  |  |
| --- | --- | --- | --- | --- | --- | --- | --- | --- | --- | --- | --- | --- | --- | --- | --- | --- | --- | --- | --- | --- | --- | --- | --- | --- | --- | --- | --- |
|  | **Male (4746)** | **Female (1938)** | **p value** | **Rural (2833)** | | **Urban (3851)** | | **p value** | **Primary or below (1749)** | | **Secondary (1338)** | |  | **High school (1558)** | | | **College/ University (1267)** | | | **Post Gradation (772)** | | | **p value** | |  |  |  |
| Antibiotics are effective against bacteria (Strongly agree/agree) | 2629 (55.4) | 923 (47.6) | <0.001 | 1433 (50.6) | | 2119 (55.0) | | 0.001 | 698 (39.9) | | 850 (63.5) | |  | 683 (43.8) | | | 788 (62.2) | | | 533 (69.0) | | | 0.001 | |  |  |  |
| Antibiotics are effective against viruses (Disagree/Strongly disagree) | 2013(42.4) | 513 (26.5) | <0.001 | 1059 (37.3) | | 1469 (38.1) | | 0.48 | 655 (37.4) | | 523 (39.1) | |  | 605 (38.8) | | | 469 (37.0) | | | 274 (35.5) | | | 0.476 | |  |  |  |
| Penicillin is an antibiotic (Strongly agree/agree) | 1500 (31.6) | 345 (17.8) | <0.001 | 753 (26.6) | | 1092 (28.4) | | 0.140 | 473(27.0) | | 363 (27.1) | |  | 443 (28.4) | | | 363 (28.7) | | | 203 (26.3) | | | 0.578 | |  |  |  |
| Paracetamol is an antibiotic (Disagree/Strongly disagree) | 1622 (34.2) | 412(21.3) | <0.001 | 825 (29.1) | | 1209 (31.4) | | 0.0046 | 541 (30.9) | | 392 (29.3) | |  | 469 (30.1) | | | 405 (32.0) | | | 227 (29.4) | | | 0.576 | |  |  |  |
| Ibuprofen is an antibiotic (Disagree/Strongly disagree) | 1486 (31.2) | 475 (24.5) | <0.001 | 790 (27.8) | | 1171 (30.4) | | 0.023 | 505 (28.9) | | 400 (29.9) | |  | 467 (30.0) | | | 368 (29.0) | | | 221 (28.6) | | | 0.921 | |  |  |  |
| If antibiotic is not taken in a way as recommended by the physician, this will increase the risk that pathogen get resistant against this antibiotic (Strongly agree/agree) | 2260 (47.6) | 792 (40.9) | <0.001 | 1256 (44.3) | | 1796 (46.6) | | 0.062 | 806 (46.1) | | 619 (46.3) | |  | 721 (46.3) | | | 567 (44.8) | | | 339 (43.9) | | | 0.746 | |  |  |  |
| Individuals who take antibiotic, regularly have a higher risk that their body is getting immuned against antibiotic. (Strongly agree/agree) | 1368 (28.8) | 428 (22.1) | <0.001 | 776 (27.4) | | 1020 (26.5) | | 0.410 | 485 (27.7) | | 344 (25.7) | |  | 416 (26.7) | | | 350 (27.6) | | | 201 (26.0) | | | 0.384 | |  |  |  |
| **Statement** | **Marital Status** | |  | **Monthly Household income (PKR)** | | | | | | **Province** | | | | | | | | | | | | |  | |  |  |  |
|  | **Single (3439)** | **Married (3245)** | **p value** | **<15000 (2398)** | **15000-30000 (2099)** | | **30000-50000 (1047)** | **>50000 (1140)** | | | **p value** | **Punjab (2431)** | | | **Sindh (2009)** | | | **KPK (1325)** | | | **Baluchistan (919) p value** | | | | |  | |
| Antibiotics are effective against bacteria (Strongly agree/agree) | 1855 (53.9) | 1697 (52.3) | 0.178 | 1300(54.2) | 1084 (51.6) | | 541 (51.7) | 627 (55.0) | | | 0.140 | 1396 (57.4) | | | | 1130 (56.2) | | | 589 (44.5) | | | 437 (47.6) | | <0.001 | | |  |
| Antibiotics are effective against viruses (Strongly agree/agree) | 1294 (37.6) | 1232 (38.9) | 0.775 | 907 (37.8) | 812 (38.7) | | 404 (38.6) | 403 (35.4) | | | 0.274 | 1231 (50.6) | | | 638 (31.8) | | | | 375 (28.3) | | | 282 (30.7) | | <0.001 | | |  |
| Penicillin is an antibiotic (Strongly agree/agree) | 942 (27.4) | 903 (27.8) | 0.543 | 632 (26.4) | 607 (28.9) | | 299 (28.5) | 308 (27.0) | | | 0.421 | 905 (37.2) | | | 468 (23.3) | | | | 277(20.9) | | | 195 (21.2) | | <0.001 | | |  |
| Paracetamol is an antibiotic (Strongly agree/agree) | 1045 (30.4) | 989 (30.5) | 0.93 | 670 (27.9) | 722 (34.4) | | 309 (29.5) | 333 (29.2) | | | <0.001 | 968 (40.6) | | | 537(26.7) | | | | 312 (23.5) | | | 199 (21.7) | | <0.001 | | |  |
| Ibuprofen is an antibiotic (Strongly agree/agree) | 999 (29.0) | 962 (28.6) | 0.592 | 709 (29.6) | 613 (29.2) | | 313 (29.9) | 326 (28.6) | | | 0.911 | 846 (34.8) | | | 558(27.8) | | | | 329 (24.8) | | | 228 (24.8) | | <0.001 | | |  |
| If antibiotic is not taken in a way as recommended by the physician, this will increase the risk that pathogen get resistant against this antibiotic (Strongly agree/agree) | 1538 (44.7) | 1514 (46.7) | 0.112 | 1101 (45.9) | 981 (46.7) | | 469 (44.8) | 501 (43.9) | | | 0.439 | 1156 (47.6) | | | 913 (45.4) | | | | 565 (42.6) | | | 418 (45.5) | | 0.032 | | |  |
| Individuals who take antibiotic, regularly have a higher risk that their body is getting immuned against antibiotic. (Strongly agree/agree) | 916 (26.6) | 880 (27.1) | 0.656 | 632 (26.4) | 555 (26.4) | | 301 (28.7) | 308 (27.0) | | | 0.491 | 791 (32.5) | | | 501 (23.9) | | | | 302 (22.8) | | | 202 (22.0) | | <0.001 | | |  |

| **Table S3 Distribution of Attitudes regarding antibiotic use according to general characteristics of the study population** | | | | | | | | | | | | | | | | | |  |  |
| --- | --- | --- | --- | --- | --- | --- | --- | --- | --- | --- | --- | --- | --- | --- | --- | --- | --- | --- | --- |
| **Statement** | **Gender** | | |  | **Residence** | |  | | |  | | **Education** | | | | | |  | |
|  | **Male (4746)** | **Female (1938)** | | **p value** | **Rural (2833)** | |  | **Urban (3851)** | | **p value** | | **Primary or below (1749)** | **Secondary (1338)** | **High school (1558)** | | **College/ University (1267)** | **Post Gradation (772)** | **p value** | |
| I have already asked my physician for a prescription of antibiotic due to cold (Disagree/Strongly disagree) | 3128 (65.9) | 1190 (61.4) | | 0.001 | 1822 (64.3) | |  | 2496 (64.8) | | 0.678 | | 1119 (64.0) | 855 (63.9) | 1026 (65.9) | | 799 (63.1) | 519 (67.2) | 0.259 | |
| I keep antibiotics at home and take it when required (Disagree/Strongly disagree) | 1690(35.6) | 703 (36.3) | | 0.607 | 1966 (69.4) | |  | 2882 (73.2) | | 0.162 | | 618 (35.3) | 492 (36.8) | 552 (35.4) | | 448 (35.4) | 283 (36.7) | 0.884 | |
| I stop taking the antibiotics when I feel better (Disagree/Strongly disagree) | 1807 (38.1) | 742 (38.3) | | 0.871 | 1077 (38.0) | |  | 1472 (38.2) | | 0.863 | | 674 (38.5) | 513 (38.3) | 587 (37.7) | | 478 (37.7) | 297 (38.5) | 0.98 | |
| If a family member is ill, I share my antibiotics with him/her (Disagree/Strongly disagree) | 2268 (47.8) | 916 (47.3) | | 0.698 | 1332 (47.6) | |  | 1852(48.1) | | 0.385 | | 833 (47.6) | 636 (47.5) | 734 (47.1) | | 599 (47.3) | 382 (49.5) | 0.862 | |
| I do not take antibiotics in general (Strongly agree/agree) | 1137 (24.0) | 448 (23.1) | | 0.464 | 667 (23.5) | |  | 1836(47.6) | | 0.001 | | 401 (22.9) | 310 (23.2) | 388 (24.9) | | 310 (24.5) | 176 (22.8) | 0.593 | |
| I am concerned about the development of antibiotic resistance (Strongly agree/agree) | 1897 (39.6) | 720 (37.2) | | 0.063 | 1073 (37.9) | |  | 3052 (79.2) | | 0.001 | | 709 (40.5) | 478 (35.7) | 600 (38.5) | | 505 (39.9) | 307 (39.8) | 0.075 | |
| **Statement** | **Marital Status** | | | | **Monthly Household income (PKR)** | | | | | | | | **Province** | | | | |  | |
|  | **Single (3439)** | | **Married (3245)** | **p value** | **<15000 (2398)** | **15000-30000 (2099)** | | **30000-50000 (1047)** | **>50000 (1140)** | | **p value** | | **Punjab (2431)** | **Sindh (2009)** | **KPK (1325)** | | **Baluchistan (919)** | **p value** |  |
| I have already asked my physician for a prescription of antibiotic due to cold (Disagree/Strongly disagree) | 2221 (64.6) | | 2097 (64.6) | 0.90 | 1621 (67.6) | 1256 (59.8) | | 717 (68.5) | 724 (63.5) | | <0.001 | | 1565 (64.4) | 1326(66.0) | 834 (62.9) | | 593 (64.5) | 0.337 |  |
| I keep antibiotics at home and take it when required (Disagree/Strongly disagree) | 1224 (35.6) | | 1168 (36.0) | 0.712 | 1722 (72.0) | 1490 (71.0) | | 748 (71.4) | 826 (72.4) | | 0.97 | | 867 (35.7) | 727 (36.2) | 470 (35.5) | | 329 (35.8) | 0.967 |  |
| I stop taking the antibiotics when I feel better (Disagree/Strongly disagree) | 1293 (37.6) | | 1256 (38.7) | 0.351 | 1756 (73.2) | 1620 (77.6) | | 798 (76.2) | 924 (81.0) | | 0.151 | | 925 (38.1) | 772 (38.4) | 513 (38.7) | | 339 (36.9) | 0.831 |  |
| If a family member is ill , I share my antibiotics with him/her (Disagree/Strongly disagree) | 1607 (47.6) | | 1577 (48.6) | 0.126 | 1158 (48.3) | 973 (46.4) | | 511 (48.8) | 542 (47.8) | | 0.499 | | 1149 (47.3) | 962 (47.9) | 623 (47.0) | | 450 (49.0) | 0.792 |  |
| I do not take antibiotics in general (Strongly agree/agree) | 741 (21.5) | | 844 (26.0) | <0.001 | 878 (36.6) | 810 (38.6) | | 399 (38.1) | 462 (40.5) | | 0.151 | | 574 (23.6) | 462 (23.0) | 321 (24.2) | | 228 (24.8) | 0.708 |  |
| I am concerned about the development of antibiotic resistance (Strongly agree/agree) | 1332 (38.7) | | 1267 (39.0) | 0.793 | 872(36.4) | 877 (41.8) | | 429(41.0) | 421(36.9) | | 0.001 | | 997 (41.0) | 779 (38.8) | 497 (37.5) | | 326 (35.5) | 0.017 |  |

**Table S4: Distribution of MDR pathogen knowledge according to general characteristics of the study population**

| **Statement** | **Gender** | | | **Locality** | | | | **Monthly Household income** | | | | | | | **Marital Status** | | | | |  |  |
| --- | --- | --- | --- | --- | --- | --- | --- | --- | --- | --- | --- | --- | --- | --- | --- | --- | --- | --- | --- | --- | --- |
|  | **Male (4746)** | **Female (1938)** | **p value** | **Rural (2833)** | | **Urban (3851)** | **p value** | **<15000 (2398)** | | **15000-30000 (2099)** | | **30000-50000 (1047)** | **>50000 (1140)** | **p value** | **Single (3439)** | | **Married (3245)** | | **p value** |  |  |
| You can only acquire multidrug-resistant pathogens in hospital (Disagree/Strongly Disagree) | 1563 (32.9) | 623 (32.1) | 0.533 | 879 (31.0) | | 1307 (33.9) | 0.001 | 792 (30.4) | | 681 (32.4) | | 345 (32.9) | 368 (32.3) | 0.90 | 1147 (33.3) | | 1039 (32.0) | | 0.063 |  |  |
| Multidrug-resistant pathogens cannot be treated (Disagree/Strongly Disagree) | 1792 (37.8) | 673 (34.7) | <0.001 | 1018 (35.9) | | 1447 (37.6) | <0.001 | 894 (37.3) | | 771 (36.7) | | 372 (35.5) | 482 (42.3) | 0.889 | 1270 (36.9) | | 1195 (36.8) | | 0.041 |  |  |
| I stop taking antibiotic immediately as soon as I feel better, I contribute to avoiding multidrug resistant pathogens (Disagree/Strongly Disagree) | 2076 (47.3) | 964 (49.7) | <0.001 | 1257 (44.4) | | 1783 (46.3) | 0.003 | 1098 (45.8) | | 952 (45.3) | | 475 (45.4) | 515 (45.2) | 0.929 | 1550 (45.1) | | 1490 (45.9) | | 0.88 |  |  |
| As long as multidrug resistant pathogens are only on my skin or mucous membrane it is not dangerous for me(Disagree/Strongly Disagree) | 2063 (43.5) | 760 (39.2) | <0.001 | 1169 (41.3) | | 1631 (42.3) | <0.001 | 1016 (42.4) | | 884 (42.1) | | 423 (40.4) | 477 (42.2) | 0.60 | 1447 (42.1) | | 1353 (41.7) | | 0.78 |  |  |
| **Statement** | **Education** | | | | | |  | **Province** | | | | | |  |  | |  | |  |  |  |
|  | **Primary or below (1749)** | **Secondary (1338)** | **High school (1558)** | | **College/University (1267)** | | | **Post Gradation (772)** | **P value** | | **Punjab (2431)** | | **Sindh (2009)** | **KPK (1325)** | | **Baluchistan (919)** | | **p value** |  |  |  |
| You can only acquire multidrug-resistant pathogens in hospital(Disagree/Strongly Disagree) | 565 (32.3) | 458 (34.2) | 508 (32.6) | | 507 (40.0) | | | 252 (32.6) | 0.90 | | 865 (35.6) | | 623 (31.0) | 421 (31.8) | | 277 (30.1) | | <0.001 |  |  |  |
| Multidrug-resistant pathogens cannot be treated (Disagree/Strongly Disagree) | 644 (36.8) | 519 (38.8) | 575 (37.0) | | 443 (35.0) | | | 284 (36.8) | 0.89 | | 967 (39.8) | | 697 (34.7) | 468 (35.3) | | 333 (36.2) | | <0.001 |  |  |  |
| I stop taking antibiotic immediately as soon as I feel better, I contribute to avoiding multidrug resistant pathogens (strongly Agree/Agree) | 784 (44.8) | 607 (45.4) | 718 (46.1) | | 574 (45.4) | | | 357 (46.2) | 0.79 | | 1191 (49.0) | | 686 (34.1) | 573 (43.2) | | 392 (42.6) | | <0.001 |  |  |  |
| As long as multidrug resistant pathogens are only on my skin or mucous membrane it is not dangerous for me (Disagree/Strongly Disagree) | 718 (41.1) | 470 (35.1) | 655 (42.0) | | 537 (42.4) | | | 320 (41.5) | 0.78 | | 1083 (44.5) | | 798 (39.7) | 535 (40.4) | | 384 (41.8) | | <0.001 |  |  |  |
